# Radiotherapy combined with DEB-BACE for locally advanced lung cancer with obstructive atelectasis following first-line treatment failure: case report

**DOI:** 10.3389/fmed.2026.1805214

**Published:** 2026-04-21

**Authors:** Bin Shen, Yunfeng Guan, Xiaolu Mao

**Affiliations:** 1 Department of Pulmonary Vascular Center, Huzhou Central Hospital, Fifth School of Clinical Medicine of Zhejiang Chinese Medical University, Huzhou, China; 2 Department of Interventional Therapy, Huzhou Central Hospital, Fifth School of Clinical Medicine of Zhejiang Chinese Medical University, Huzhou, China; 3 Huzhou Central Hospital, Affiliated Central Hospital of Huzhou University, Huzhou, China; 4 Department of Radiation Oncology, Huzhou Central Hospital, Fifth School of Clinical Medicine of Zhejiang Chinese Medical University, Huzhou, China

**Keywords:** DEB-BACE, interventional therapy, non-small cell lung cancer (NSCLC), obstructive atelectasis, radiotherapy

## Abstract

For patients with locally advanced non-small cell lung cancer (NSCLC) who are intolerant to concurrent chemoradiotherapy, sequential chemotherapy followed by radiotherapy is a standard alternative treatment. However, disease progression after first-line chemotherapy results in poor prognosis, and radiotherapy alone often fails to achieve ideal tumor control. Notably, these patients exhibit a higher incidence of obstructive atelectasis, which further worsens clinical outcomes. This study reports two unique cases of locally advanced NSCLC with obstructive atelectasis after first-line chemotherapy failure. Both patients received combined therapy with radiotherapy and drug-eluting bead bronchial artery chemoembolization (DEB-BACE). Results demonstrated preliminary control of atelectasis in both patients, followed by long-term remission post-treatment. These findings suggest that radiotherapy combined with DEB-BACE may be a promising potential strategy for locally advanced NSCLC patients accompanied by atelectasis.

## Introduction

For patients with locally advanced non-small cell lung cancer (NSCLC), sequential radiotherapy following chemotherapy serves as the standard alternative treatment option for those unable to tolerate concurrent chemoradiotherapy (1). However, disease progression after first-line chemotherapy in these patients is associated with significantly worse clinical outcomes, and the effectiveness of subsequent radiotherapy is often limited (2). It is noteworthy that these patients have a high incidence of atelectasis, which is frequently induced by bronchial obstruction. Moreover, atelectasis itself is an unfavorable factor affecting prognosis (3). Thus, managing locally advanced NSCLC with atelectasis after first-line chemotherapy failure remains a clinical challenge, with limited treatment options and unfavorable outcomes.

Drug-eluting bead bronchial artery chemoembolization (DEB-BACE), as a novel interventional technique, has been increasingly investigated and applied in the treatment of both newly diagnosed and advanced NSCLC patients (4, 5). This approach achieves sustained local release of chemotherapeutic agents by embolizing tumor-feeding arteries with drug-eluting beads, thereby leveraging the dual antitumor effects of “ischemia-hypoxia combined with chemotherapy” (6). It offers the advantages of precise local efficacy and minimal systemic adverse effects, showing particularly notable effectiveness against atelectasis by effectively improving the rate of dyspnea relief and lung re-expansion. For locally advanced NSCLC patients with atelectasis, intolerance to intravenous chemotherapy, and progression after first-line chemotherapy, the combination of radiotherapy and DEB-BACE may represent a promising treatment strategy. Given the limited published experience with this specific combination, this paper presents a preliminary report of its exploratory application in two such patients.

## Case 1

**Figure 1:**
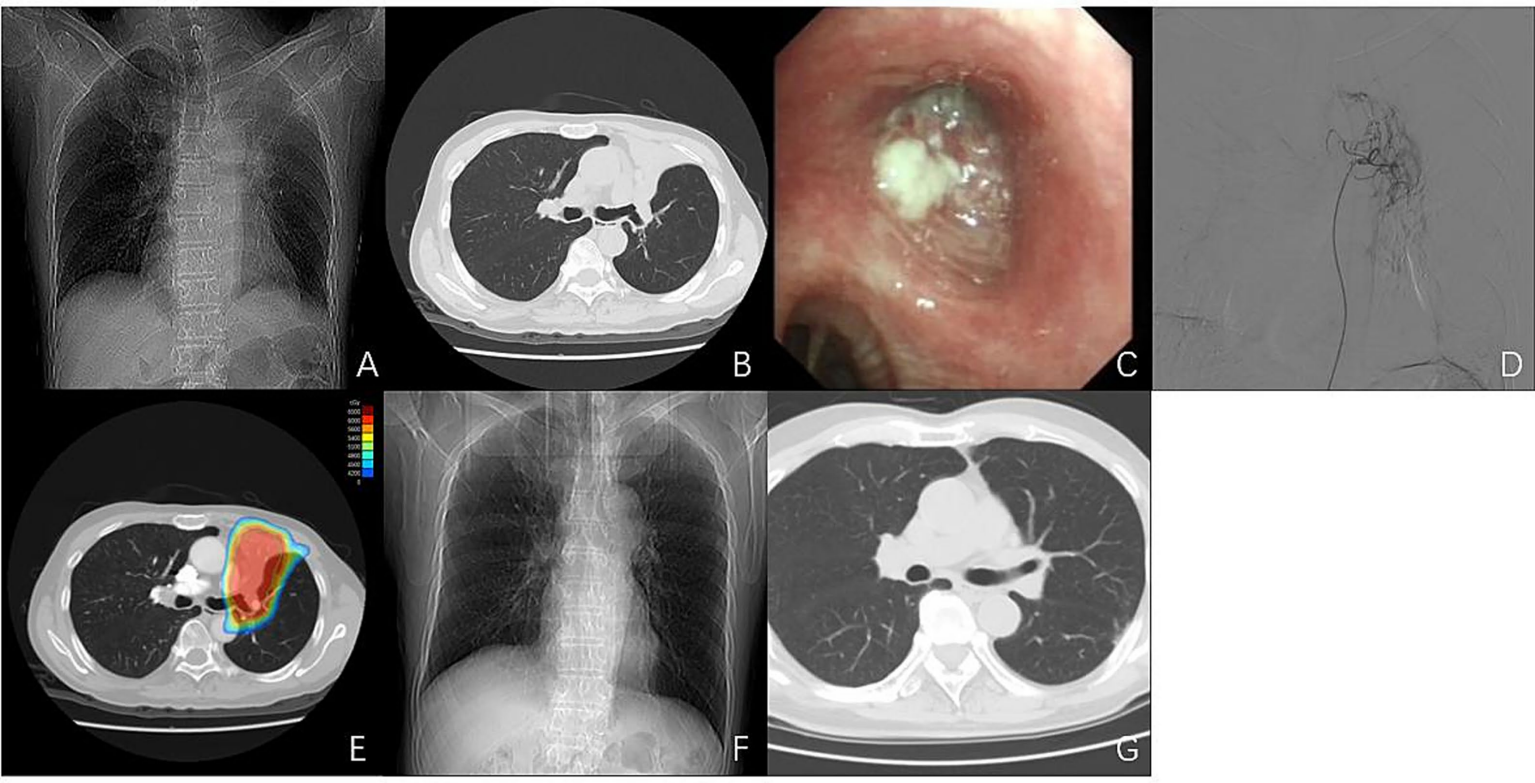
Imaging, endoscopic, DSA findings, and dose distribution in Case 1. **(A,B)** Chest CT demonstrates occlusion of the left upper lobe bronchus with atelectasis of the left upper lobe. **(C)** Bronchoscopy reveals a neoplasm obstructing the orifice of the left upper lobe. **(D)** DSA demonstrating hypertrophic and tortuous tumor-feeding arteries with dense tumor staining. **(E)** Dose distribution of volumetric modulated arc therapy (VMAT) for the patient. **(F,G)** Follow-up chest CT 1 month after combined treatment, showing complete tumor regression and re-expansion of the left upper lobe.

A 65-year-old male with a history of hypertension was admitted in November 2019 due to a 4-month history of cough, accompanied by chest tightness and shortness of breath after physical activity. He was diagnosed with squamous cell carcinoma of the left lung. Immunohistochemistry results were as follows: CK5/6 (+), P40 (+), TTF-1 (−), and Ki-67 (positive in approximately 40% of tumor cells). The tumor proportion score (TPS) for PD-L1 expression was approximately 1%. The clinical stage was cT2N0M0 (stage IB). Since the patient could not tolerate left pneumonectomy, he received two cycles of neoadjuvant chemotherapy with the TC regimen (albumin-bound paclitaxel plus carboplatin). Subsequent follow-up indicated disease progression with worsened chest tightness. Physical examination showed a performance status (PS) score of 2, diminished breath sounds in the left lung, and a modified Medical Research Council (mMRC) grade of 3. Chest CT scan showed obstruction of the left upper lobe bronchus and complete atelectasis of the left upper lobe (Figures 1A,B). Bronchoscopy demonstrated a neoplasm obstructing the left upper lobe orifice (Figure 1C). Due to the patient’s poor PS score, which made concurrent chemoradiotherapy intolerable, and the expected limited efficacy of radiotherapy alone, the multidisciplinary team (MDT) decided to implement a combined therapy of DEB-BACE and radiotherapy.

DEB-BACE was performed first. Intraoperative digital subtraction angiography (DSA) clearly demonstrated hypertrophic and tortuous tumor-feeding arteries with dense tumor staining (Figure 1D). The intervention utilized HepaSphere^®^ microspheres (50–100 μm), where irinotecan (100 mg) was slowly loaded over 30 min. The drug-eluting microspheres were then slowly infused until complete blood flow stasis was achieved in the tumor vasculature. Concurrently, volumetric modulated arc therapy (VMAT) was initiated, delivering 66 Gy in 30 fractions to the gross tumor volume (GTV) and 60 Gy in 30 fractions to the planning target volume (PTV) (Figure 1E).

One week after radiotherapy, the patient’s dyspnea had significantly improved, with the mMRC grade decreasing to 1. Follow-up chest CT 1 month after radiotherapy completion demonstrated complete remission of the left upper hilar lesion and full re-expansion of the left upper lobe (Figures 1F,G). The patient subsequently received 30 cycles of maintenance therapy with durvalumab, achieving a progression-free survival (PFS) of 68 months. At the last follow-up, the patient maintained a durable complete response with no evidence of disease progression. According to Common Terminology Criteria for Adverse Events (CTCAE) version 5.0, treatment-related toxicity was limited to grade 2 myelosuppression, which was effectively managed with appropriate supportive treatment.

## Case 2

**Figure 2:**
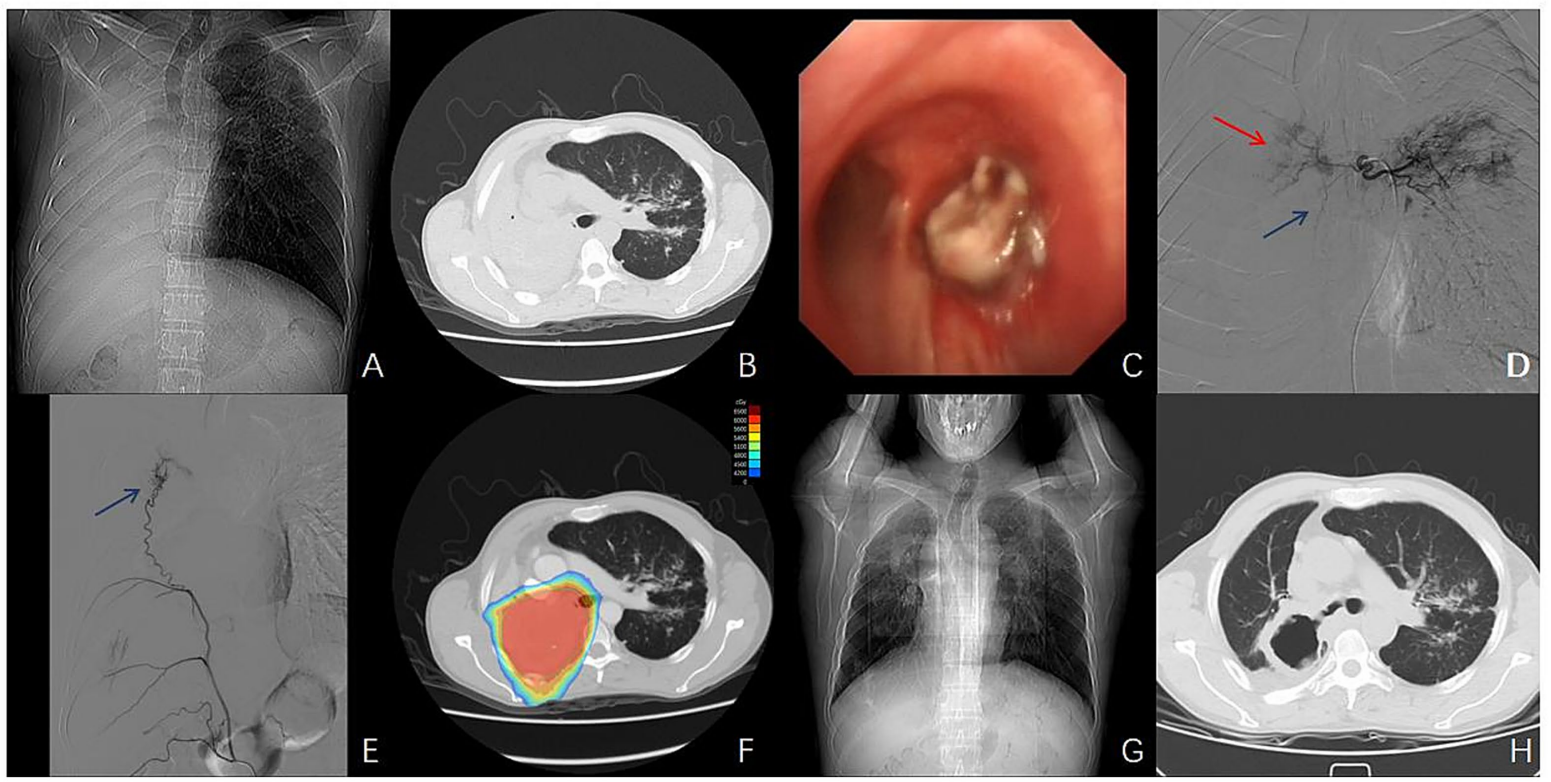
Imaging, endoscopic, DSA findings, and dose distribution in Case 2. **(A,B)** Chest CT demonstrates complete atelectasis of the right lung and occlusion of the right main bronchus. **(C)** Bronchoscopy reveals a neoplasm completely obstructing the orifice of the right main bronchus, with mucosal swelling and infiltration extending to the carina. **(D)** DSA demonstrating tumor staining in the right branch of the common trunk of the left and right bronchial arteries, with an absence of staining at the inferior tumor margin. **(E)** Localized tumor staining supplying the inferior margin is observed in the distal branches of the right inferior phrenic artery. **(F)** Dose distribution of volumetric modulated arc therapy (VMAT) for the patient. **(G,H)** Follow-up chest CT at 3 weeks after combined treatment indicates tumor reduction with central cavitary necrosis and re-expansion of the right upper and middle lobes.

A 56-year-old male was admitted in December 2019 with a diagnosis of lung cancer confirmed approximately 20 days earlier. Chief complaints included cough with sputum production, hemoptysis, and chest tightness. He was diagnosed with squamous cell carcinoma of the right lung, clinically staged as cT4N1M0 (stage IIIA). After two cycles of chemotherapy with the GC regimen (gemcitabine and carboplatin), disease progression occurred, manifested as exertional dyspnea and chest tightness. Physical examination revealed absent breath sounds over the right lung, with an mMRC grade of 4. Chest CT demonstrated malignant tumor in the right upper lobe accompanied by increased right lung atelectasis (Figures 2A,B). Bronchoscopy identified a neoplasm completely obstructing the orifice of the right main bronchus, with mucosal swelling and infiltration extending to the carina (Figure 2C). The patient had previously experienced grade IV myelosuppression with severe infection following chemotherapy, and his current performance status (PS) score was 2, unable to tolerate further concurrent intravenous chemotherapy. Radiotherapy alone offered limited tumor control. After an urgent multidisciplinary team (MDT) consultation, a treatment regimen combining DEB-BACE with radiotherapy was initiated.

DEB-BACE was initially performed. Angiography demonstrated distal tumor staining in the right branch of the common trunk of the left and right bronchial arteries, with an absence of staining at the inferior tumor margin (Figure 2D). Localized tumor staining supplying the inferior margin was observed in the distal branches of the right inferior phrenic artery (Figure 2E). DEB-BACE was performed using an identical drug-loading protocol as in Case 1, with irinotecan (100 mg) loaded onto HepaSphere^®^ microspheres. Radiotherapy commenced 3 days post-procedure with a PTV dose of 60 Gy in 30 fractions (Figure 2F).

One week after radiotherapy, the patient showed significant improvement in dyspnea. Two weeks later, the mMRC grade was assessed as 2. Chest CT scan 3 weeks after the completion of radiotherapy showed shrinkage and cavitary necrosis of the right lung mass, along with re-expansion of the right lung (Figures 2G,H), consistent with a partial response (PR). Following the combination therapy of DEB-BACE and radiotherapy, the patient did not receive further systemic anti-tumor treatment. The last follow-up was conducted in April 2021, at which time the disease was assessed as stable (SD) with no evidence of progression. The patient later died at an outside hospital due to pulmonary infection. The progression-free survival (PFS) was 9 months. According to CTCAE version 5.0 criteria, the treatment-related toxicity was limited to grade 2 myelosuppression, which was effectively managed with appropriate supportive care.

## Discussion

Lung cancer is one of the most prevalent and deadliest malignancies worldwide (7). Non-small cell lung cancer (NSCLC) accounts for approximately 80–85% of all cases, and the majority of patients are diagnosed at a locally advanced or advanced stage. For patients in poor general condition who cannot tolerate concurrent chemoradiotherapy, sequential chemotherapy followed by radiotherapy serves as the standard alternative treatment option. However, after first-line chemotherapy failure, about 40–55% of patients develop obstructive atelectasis due to local tumor progression (3). These patients often present with severe respiratory impairment, persistent infections, and poor performance status, rendering them unable to tolerate systemic chemotherapy. Conventional palliative radiotherapy mainly acts by inducing DNA damage and delayed tumor cell death, which often fails to rapidly relieve symptoms in cases of acute airway obstruction. Its efficacy is also limited in large tumor volumes or under hypoxic conditions, which are common in atelectatic areas. The local response rate is relatively low, with only about 23% of patients achieve tumor control and lung re-expansion after treatment, making it difficult to rapidly alleviate emergent symptoms or prolong survival (8). Some patients even have to discontinue therapy due to worsening clinical symptoms.

Currently, bronchoscopic endobronchial interventional therapy has become a primary approach for managing malignant central airway stenosis in patients who are inoperable or decline surgery, as it can rapidly relieve central airway obstruction through mechanical means (9). However, the procedure carries various risks of complications, among which massive airway bleeding is the most common and critical. Once it occurs, airway obstruction and asphyxia may rapidly ensue, posing an immediate life-threat if not managed promptly. Other complications, such as pneumothorax, mediastinal emphysema, airway perforation, and aggravated infection (10), further increase treatment risks and physical burden on patients. For patients with obstructive atelectasis who have poor performance status and compromised baseline respiratory function, their tolerance for complications is even lower, which may lead to treatment interruption and worse prognosis. Additionally, bronchoscopic intervention primarily addresses intraluminal stenosis and does not directly target the extraluminal tumor burden causing compression. This may limit its long-term control efficacy and necessitate repeated procedures.

Therefore, there is an urgent need to explore an effective treatment strategy that offers both rapid local efficacy and sustained tumor control for such advanced NSCLC patients presenting with acute complications and poor performance status.

DEB-BACE is an interventional technique originally developed for the treatment of hepatocellular carcinoma, and its effectiveness and safety have been confirmed by multiple clinical studies (11, 12). In recent years, this technique has been gradually applied in the treatment of lung tumors (13–15). DEB-BACE involves the embolization of tumor-feeding arteries with drug-eluting microspheres that slowly release chemotherapeutic agents, effectively reducing tumor volume, alleviating airway compression, and thereby achieving lung re-expansion and local tumor control (16). The drug-eluting microspheres exhibit high affinity for chemotherapeutic drugs and can stably anchor within the microvasculature of the tumor region (17), enabling the slow release of drugs into the surrounding tumor tissue. This leads to a relatively high local drug concentration within a short period, leading to efficient tumor kill. Due to this mechanism, effective local drug concentration can be maintained with a lower administered dose, making it suitable for patients in poor systemic condition who cannot tolerate high-dose intravenous chemotherapy. Compared with conventional intravenous chemotherapy, DEB-BACE offers the distinct advantages of higher local drug concentration and lower systemic toxicity. It can achieve favorable antitumor effects while rapidly relieving clinical symptoms, creating favorable conditions for subsequent comprehensive treatment (18).

DEB-BACE enables highly selective sustained delivery of chemotherapeutic drugs within the tumor and permanent embolization of the feeding vessels, which can effectively control the hypervascular core region of the tumor (19). The therapeutic potential of this technique in advanced lung cancer has been preliminarily confirmed. A prospective study conducted by Xu Ma et al. enrolled 20 patients with locally advanced or advanced lung cancer who developed obstructive atelectasis after failure of conventional treatment. All patients received DEB-BACE therapy. Follow-up results showed that 16 patients achieved partial response (PR) and 1 had stable disease (SD), with an objective response rate (ORR) of 80% and a disease control rate (DCR) of 85%. The lung re-expansion rate was 80% (16/20). The improvement rates of dyspnea at 1 week and 1 month after treatment were 85 and 80%, respectively (*p* < 0.0001, *p* < 0.0001) (20). This provides a promising therapeutic approach for alleviating atelectasis and dyspnea symptoms.

This study reports two cases of locally advanced lung cancer with obstructive atelectasis, in which the patients were unable to tolerate intravenous chemotherapy due to myelosuppression after prior chemotherapy or poor PS scores. Given the low symptom remission rate and potential life-threatening risks associated with radiotherapy alone, our multidisciplinary team explored a combined regimen of radiotherapy and DEB-BACE. The treatment sequence was strategically designed as DEB-BACE followed by radiotherapy, a decision based on both clinical urgency and the physiological goal of optimizing treatment efficacy. In the context of acute obstructive atelectasis, DEB-BACE was prioritized to achieve rapid tumor cytoreduction and restore airway patency, which significantly improved the patients’ respiratory function and performance status, making them better able to tolerate the subsequent daily radiotherapy course. Physiologically, the initial intervention with DEB-BACE serves to alleviate intratumoral hypoxia by improving microcirculation, thereby reducing radiotherapy resistance and allowing the high local concentration of chemotherapeutic agents to act as a radiosensitizer. Furthermore, initiating treatment with DEB-BACE facilitates more precise radiotherapy planning; by inducing lung re-expansion first, the multidisciplinary team was able to perform more accurate target delineation (GTV and PTV), ensuring that the radiation dose was delivered effectively to the tumor while minimizing exposure to healthy lung tissue that was previously collapsed. This “debulking-first” approach represents an active palliative strategy specifically designed for medically fragile patients with severe respiratory compromise who might otherwise experience treatment failure or interruption due to progressive airway obstruction. Subsequent radiotherapy then provides coverage of the primary lesion and potential microscopic infiltrates by damaging tumor cell DNA, inhibiting cell division, and inducing cell death (21). This sequence is intended to harness a potential synergy: DEB-BACE achieves rapid tumor reduction and symptom relief, creating a more favorable target and potentially enhancing the effects of radiotherapy, which subsequently provides comprehensive local control.

In this study, we selected HepaSphere^®^ microspheres (dry size 50–100 μm) loaded with 100 mg of irinotecan, a combination chosen for its unique biophysical properties and pharmacological synergy. Unlike pre-swollen microspheres such as CalliSpheres^®^, HepaSphere^®^ is a dry, swellable microsphere that expands upon contact with aqueous solutions, which allows for deeper penetration into the distal tumor-feeding microvasculature before achieving complete embolization (22). This distal occlusion is particularly effective for rapidly reducing tumor volume to alleviate bronchial obstruction and facilitate lung re-expansion. Both patients involved in this study were resistant to the GP/PC regimen; therefore, irinotecan was subsequently selected as the loaded drug. This choice was based not only on the established antitumor activity of irinotecan in non-small cell lung cancer, but also on its potential value as a radiosensitizer, which theoretically could produce a synergistic effect with the subsequent volumetric modulated arc therapy (VMAT), thereby potentially enhancing local control of refractory lesions.

The results showed that both patients achieved favorable clinical outcomes: following the combination of DEB-BACE and radiotherapy, the tumors shrank significantly, effective lung re-expansion was achieved, and clinical remission was obtained. No disease progression was observed during long-term follow-up. Patient in Case 1 received durvalumab immunotherapy maintenance after treatment with radiotherapy combined with DEB-BACE, which may have contributed to the observed PFS of up to 68 months. The PACIFIC study demonstrated that consolidation immunotherapy with durvalumab after concurrent chemoradiotherapy significantly improves PFS in patients with unresectable stage III NSCLC (23). We hypothesize that the local tumor reduction induced by radiotherapy combined with DEB-BACE, along with its potential immunogenic effects, may have created a more favorable microenvironment for subsequent immune checkpoint inhibitor therapy. This could be one of the reasons for the long-term disease control observed in this case, though this requires validation in larger prospective studies.

Regarding safety, no patient experienced grade ≥3 toxicity, indicating that this combination regimen has a manageable safety profile. However, the potential risks of this combined therapy still warrant comprehensive discussion. As an arterial interventional procedure, DEB-BACE itself carries inherent risks such as puncture site complications, vascular injury, non-target embolization, and post-embolization syndrome. Combining it with radiotherapy could theoretically increase the risk of toxicities, particularly radiation pneumonitis, as both modalities can affect lung tissue. The spatial and dosimetric interaction between the embolized area and the irradiated volume remains unclear and may potentially increase the likelihood of pulmonary complications. To mitigate potential pulmonary toxicity, this treatment strategy intentionally avoided the use of chemotherapeutic agents known for significant lung toxicity (e.g., gemcitabine) (24). Furthermore, a planned interval was set between DEB-BACE and the initiation of radiotherapy, and strict dose constraints for normal lung tissue were adhered to during radiotherapy planning. The absence of serious adverse events in both cases is encouraging but remains a preliminary finding. The safety of this combination, particularly regarding long-term pulmonary sequelae, needs to be carefully evaluated in larger prospective studies incorporating detailed pulmonary function tests and imaging follow-up.

The DEB-BACE technique employed in this study presents certain technical barriers and procedural limitations. The procedure itself is relatively complex and demands a high degree of embolization precision, typically requiring performance by an experienced interventional radiologist. Pre-procedural fine imaging assessment, such as thin-slice contrast-enhanced CT, is relied upon to delineate the tumor-feeding arteries. In some cases, these feeding vessels are extremely fine, which significantly increases the difficulty of superselective catheterization and complete embolization. Of particular concern is that for patients whose tumors have severely invaded the pulmonary arterial wall, rapid tumor shrinkage following DEB-BACE may potentially induce pulmonary artery rupture syndrome, leading to life-threatening massive hemorrhage (20). These technical characteristics and risks make it challenging to routinely implement the technique across all medical centers. Its broader clinical adoption is constrained by multiple factors, including equipment availability, specialized training resources, and patient selection.

The efficacy assessment in this study was primarily based on imaging examinations and improvement in clinical symptoms. While these are essential foundations for evaluating response in solid tumors, certain limitations remain. Blood-based biomarkers, particularly circulating tumor DNA (ctDNA), represent an important advancement in this field, as they can monitor tumor dynamics and treatment response with minimal invasiveness (25). However, since the treatments for the two patients in this report began in late 2019, longitudinal ctDNA monitoring was not part of our center’s routine clinical protocol at that time; therefore, relevant data were not obtained. Future studies could incorporate such blood biomarkers for dynamic monitoring, thereby effectively complementing conventional imaging-based evaluations.

Certainly, the safety of this combined treatment regimen, the optimal timing between intervention and radiotherapy, dose coordination, and long-term efficacy still require further validation through prospective clinical studies. Future research should focus on evaluating the impact of combined therapy on patients’ respiratory function, quality of life, local progression-free survival, and overall survival. Close monitoring for potentially increased toxicities is also necessary, including the overlapping or additive effects of complications such as radiation pneumonitis, esophagitis, and those related to the interventional procedure. This will provide more substantial evidence to support the broader clinical application of this therapeutic approach.

## Conclusion

For patients with locally advanced NSCLC who have obstructive atelectasis and disease progression after first-line chemotherapy, radiotherapy combined with DEB-BACE may represent a promising treatment option. However, this study is a small-sample case analysis, and its conclusions are limited. Prospective, large-sample studies are still needed to confirm the efficacy and safety of this technique.

## Data Availability

The original contributions presented in the study are included in the article/supplementary material, further inquiries can be directed to the corresponding author.
